# Serum Metabolomic Alterations and Developmental Toxicity Induced by Diquat Exposure in Pregnant Rats and Their Fetuses: A Preliminary Study

**DOI:** 10.3390/toxics14080656

**Published:** 2026-07-25

**Authors:** Xuelian Li, Ying Cai, Mengcao Liu, Guang Wang, Lina Gao

**Affiliations:** 1 Key Laboratory of Environmental Stress and Chronic Disease Control & Prevention, China Medical University, Ministry of Education, Shenyang 110122, China; xlli@cmu.edu.cn (X.L.);; 2 Department of Epidemiology, School of Public Health, China Medical University, Shenyang 110122, China; 3 Teaching Center for Basic Medical Experiment, China Medical University, Shenyang 110122, China; 4 Department of Laboratory Animal Science, China Medical University, Shenyang 110122, China; 5 Department of Forensic Analytical Toxicology, School of Forensic Medicine, China Medical University, Shenyang 110122, China

**Keywords:** diquat, reproductive and developmental toxicity, oxidative stress, metabolomics

## Abstract

The reproductive and developmental toxicity of diquat (DQ), a widely used herbicide, during pregnancy remains insufficiently characterized. This study investigated the effects of repeated DQ exposure on fetal development and maternal metabolism in pregnant Sprague–Dawley rats. The rats were administered sublethal doses of DQ (28.88 and 57.57 mg/kg), and maternal serum sex hormones, fetal skeletal development, and serum metabolomic profiles were evaluated. Compared with controls, serum estradiol levels were significantly decreased in the high-dose exposure group, whereas luteinizing hormone and follicle-stimulating hormone levels remained unchanged. Significant alterations in malondialdehyde, superoxide dismutase, and glutathione levels indicated oxidative stress in exposed rats. Severe fetal skeletal ossification deficits and developmental delays were observed, with incomplete or failed ossification of the occipital bone, sternum, caudal vertebrae, and pelvis, with litter-level incidences reaching 100%. Metabolomic analysis identified distinct serum metabolic profiles between DQ-exposed and control groups, suggesting ovarian steroidogenesis, phenylalanine metabolism, and aldosterone synthesis and secretion as key pathways affected by DQ toxicity. These findings suggest that repeated maternal exposure to DQ induces oxidative stress, disrupts metabolic and hormonal homeostasis, and causes delayed fetal skeletal development. This study provides novel evidence for understanding the reproductive and developmental toxicity of DQ and its potential metabolic mechanisms.

## 1. Introduction

**Figure 1:**
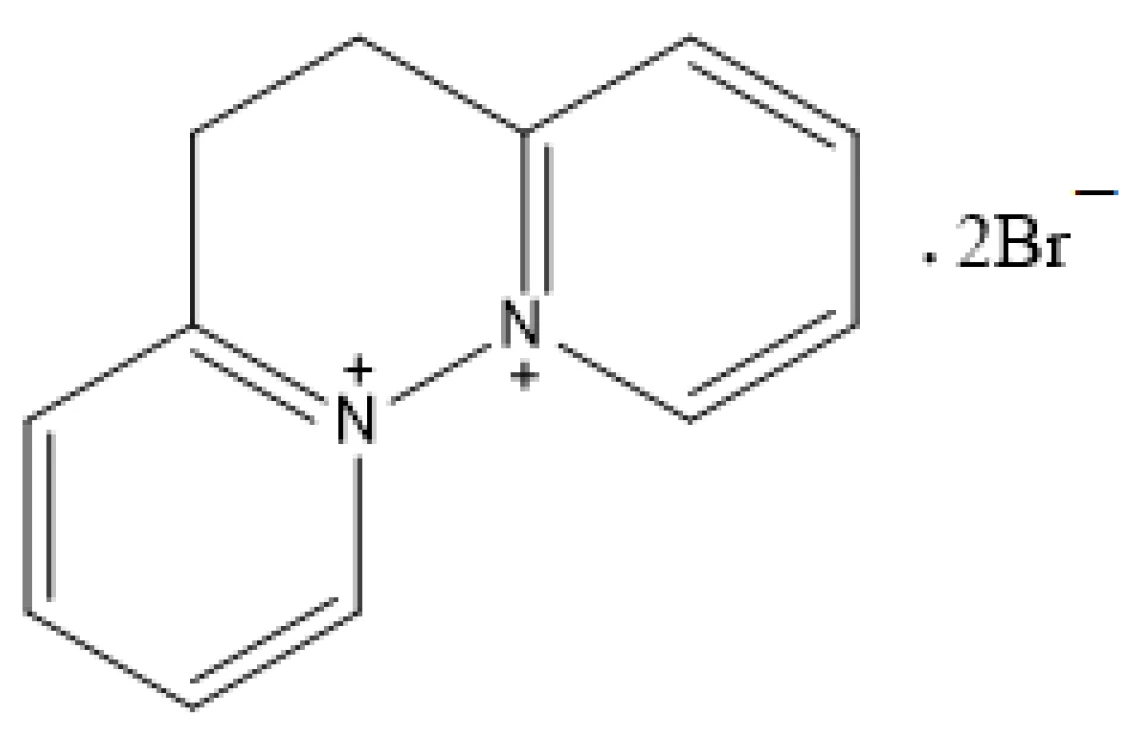
Chemical structure of diquat.

Diquat (DQ; chemical structure shown in Figure 1) is a fast-acting herbicide widely used in agriculture, horticulture, and aquatic weed control. However, its high toxicity and environmental persistence have led to severe restrictions on its use in many countries [1,2]. While environmental concentrations of DQ are typically low, they can increase substantially in areas with intensive use, raising concerns about potential human exposure [1,2,3,4]. Beyond its well-documented systemic toxicity affecting the gastrointestinal tract, kidneys, liver, lungs, and central nervous system [2,4,5,6], the potential risks of DQ exposure during pregnancy, a period of heightened vulnerability, remain critically under-explored.

Pregnancy is a particularly vulnerable period characterized by rapid physiological changes and increased susceptibility to toxins that may disrupt organogenesis and cause long-term adverse effects on offspring [7]. Despite growing concerns regarding developmental susceptibility to environmental toxicants, studies investigating DQ exposure during pregnancy remain limited and have yielded inconsistent findings. Chernoff et al. [8] reported no significant developmental effects in offspring at DQ doses that induced overt maternal toxicity, including weight loss and mortality. Zhang et al. [5] reported that DQ exposure impairs oocyte quality, induces apoptosis in follicular granulosa cells, and promotes follicular atresia, thereby leading to reproductive toxicity in female mice. In addition, studies have also shown that DQ exposure can impair bone development in mice [9], while exposure of Mallard eggs to 16 g/L DQ on day 4 of incubation resulted in malformations [10]. Furthermore, Yu et al. [6] highlighted a case of acute maternal DQ poisoning, demonstrating that DQ can cross the placental barrier, affect the fetus, induce miscarriage, and ultimately lead to maternal death.

The inconsistencies among these studies raise several critical questions that remain unresolved. First, the discordance between Chernoff et al.’s negative findings and the positive evidence from other studies may arise from differences in species sensitivity, exposure timing, or dose regimens. Second, most animal studies have focused on general reproductive outcomes (e.g., litter size, resorptions, fetal weight) rather than on structural endpoints such as skeletal ossification, which our preliminary dose-finding study suggested may be particularly sensitive to DQ.

Several studies have also raised concerns regarding the endocrine-disrupting potential of DQ [11,12]. As a potent oxidative stress inducer, DQ generates excessive reactive oxygen species, thereby interfering with endocrine functions at multiple levels. However, systematic investigations of gestational DQ exposure in mammalian models remain scarce, particularly regarding maternal endocrine perturbations and fetal structural abnormalities. To bridge this knowledge gap, the present study evaluated the impacts of prenatal DQ exposure on fetal skeletal development and maternal endocrine function, with a focus on hormonal regulation and ossification outcomes.

Among the numerous hormonal adaptations occurring during pregnancy, serum estradiol (E_2_) plays a pivotal physiological role [13]. Initially synthesized by the maternal ovaries and subsequently by the fetoplacental unit, E_2_ regulates uterine receptivity, placental vascular remodeling, and fetal growth and development [14]. Dysregulation of E_2_ homeostasis has been associated with adverse pregnancy outcomes. Given that oxidative stress directly inhibits key steroidogenic enzymes such as aromatase (CYP19A1), assessment of E_2_ levels during DQ exposure may provide mechanistic insight into endocrine disruption [15,16]. We acknowledge that other steroid hormones, including progesterone, pregnenolone, and aldosterone, also participate in pregnancy maintenance and fetal development. However, E_2_ was prioritized a priori as the primary endocrine readout because of its established sensitivity to oxidative stress, its well-characterized role in skeletal development, and its physiological predominance during the gestational period under investigation.

Fetal skeletal development is highly sensitive to in utero exposure to toxicants [17,18]. Osteogenesis involves the differentiation of mesenchymal progenitor cells into chondrocytes and osteoblasts under the regulation of multiple signaling molecules and hormones [19,20,21]. E_2_ contributes to bone development via estrogen receptor-mediated signaling [22]. Consequently, disruption of E_2_ homeostasis during critical developmental windows may alter fetal skeletal morphology. Standardized skeletal assessment therefore provides an integrated endpoint for evaluating developmental toxicity arising from hormonal and metabolic disruptions.

Metabolomics, a systems biology approach integrating genomics and proteomics, enables comprehensive characterization of metabolic alterations and provides valuable insights into toxicological mechanisms [23,24].

Taken together, the present study was designed to investigate the effects of gestational DQ exposure on maternal serum E_2_ levels and fetal skeletal development, while concurrently employing serum metabolomics to explore the underlying metabolic perturbations. We hypothesized that DQ exposure during pregnancy would disrupt E_2_ homeostasis, impair fetal skeletal development, and induce discernible alterations in the maternal serum metabolome associated with oxidative stress and endocrine dysfunction.

## 2. Materials and Methods

### 2.1. Chemicals

Diquat dibromide (98% purity) was acquired from Beijing Green Leaf Reagent Co., Ltd. (Beijing, China). Rat FSH, LH, and E_2_ ELISA kits were purchased from Shanghai Yuanju Biotechnology Center (Shanghai, China). MDA, GSH, and SOD assay kits were obtained from Elabscience Biotechnology Co., Ltd. (Wuhan, China). Additional reagents used in this study included isoflurane, absolute ethanol, potassium hydroxide, glycerol, Alizarin Red, and Bouin’s solution. All chemicals were of analytical grade.

### 2.2. Animals and Treatment

A total of 18 female Sprague–Dawley rats (11–12 weeks old, 220–250 g) and nine male rats were obtained from Beijing Huafukang Biotechnology Co., Ltd. (Beijing, China). The animals were housed under standard laboratory conditions (23–25 °C, 50–60% relative humidity, 12 h light/dark cycle, and 12–14 air changes per hour) with ad libitum access to food and water. Following a 1-week acclimation period, mating was initiated at a female:male ratio of 2:1. The female rats were paired with males daily at 7:00 p.m. and examined the following morning at 7:00 a.m. for evidence of mating. Successful mating was confirmed by the presence of a vaginal plug or sperm in vaginal smears examined under a light microscope. The day on which sperm was detected in the smear was designated as gestation day 1 (GD1).

**Figure 2:**
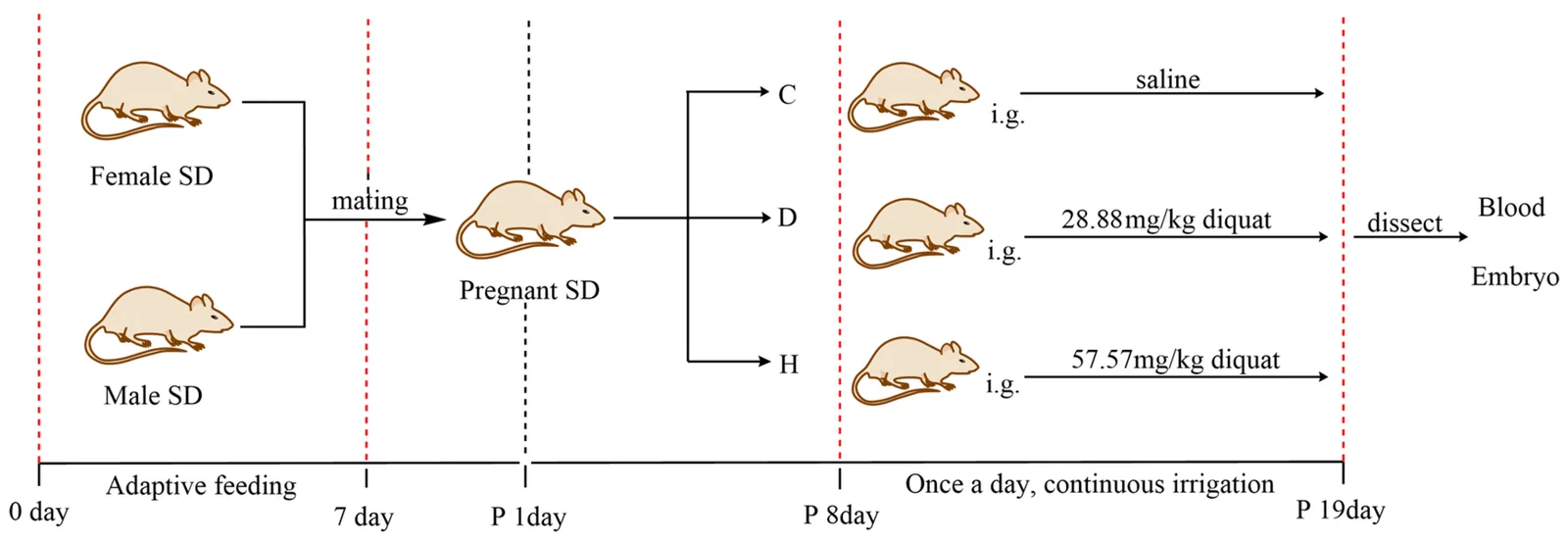
Experimental design (C, control group; D, low-dose exposure group; H, high-dose exposure group).

Upon confirmation of successful mating, the dams were weighed and assigned to the control (*n* = 6), low-dose (*n* = 5), and high-dose (*n* = 5) groups according to a random number table. Two non-pregnant female rats were excluded from the study. In preliminary dose-finding studies, administration of DQ initiated immediately after mating (GD0) resulted in complete pregnancy failure in all treated dams, as evidenced by the absence of viable implantation sites. To enable assessment of post-implantation developmental toxicity, DQ administration was therefore initiated on GD9. The animals were maintained under normal husbandry conditions until GD8, after which they received daily oral gavage treatments until GD19. The experimental design is illustrated in Figure 2. Given the widespread use of diquat dibromide and its potential for acute oral poisoning following accidental ingestion, the exposure doses were established at approximately 1/5 and 1/10 of the oral LD_50_ value in rats (280 mg/kg) [25,26], representing typical acute exposure levels reported in clinical cases. The selection of 1/10 LD_50_ serves as a subacute exposure starting point to detect early biochemical effects, while the 1/5 LD_50_, being a 2-fold higher dose, was chosen to establish a clear dose–response relationship. Both doses are substantially lower than the acute lethal dose, thereby minimizing non-specific systemic toxicity and allowing for the specific evaluation of diquat’s impact on reproductive and developmental toxicity. The low-dose and high-dose groups received aqueous DQ solutions at daily doses of 28.88 and 57.75 mg/kg, respectively, while the control group received an equivalent volume of saline. All the experimental procedures were approved by the Animal Ethics Committee of China Medical University (Approval No. 2022353).

### 2.3. Sample Collection

On GD19, all pregnant rats were euthanized by decapitation under isoflurane anesthesia. In brief, rats were placed in an induction chamber containing 4–5% isoflurane in 100% oxygen until loss of consciousness and absence of pedal withdrawal reflex. Anesthesia was maintained with 2–2.5% isoflurane delivered via nose cone. After confirmation of a surgical plane of anesthesia (absence of response to toe pinch), decapitation was performed using a guillotine. Blood samples were collected and centrifuged at 3000 rpm and 4 °C for 15 min to obtain serum, which was stored at −80 °C until analysis. The uteri were subsequently opened and the fetuses were counted. Following an initial visual examination, the fetuses were preserved systematically: odd-numbered fetuses were fixed in Bouin’s solution for histological studies, whereas even-numbered fetuses were preserved in anhydrous ethanol for further morphological analysis.

### 2.4. Measurement of Serum Sex Hormone Levels

Serum levels of FSH, LH, and E_2_ were measured using ELISA kits according to the manufacturer’s instructions. The mean value of technical duplicates for each sample was used as a single data point for statistical analyses. Optical density (OD) was determined at 450 nm using a microplate reader. Standard curves were generated from known standard concentrations and corresponding OD values to calculate hormone concentrations (FSH: detection range = 0.625–40 mIU/mL, limit of detection (LOD) = 0.31 mIU/mL; LH: detection range = 1.56–800 mIU/mL, LOD = 0.78 mIU/mL; E_2_: detection range = 2.86–183.5 pmol/L, LOD = 1.43 pmol/L). The coefficients of variation (CVs) for all assays were <10%.

### 2.5. Determination of Malondialdehyde, Superoxide Dismutase, and Glutathione Levels

Serum levels of MDA, SOD, and GSH were evaluated using commercial assay kits, according to the manufacturer’s instructions.

### 2.6. Assessment of Reproductive Performance and Fetal Morphology

On GD19, following isoflurane anesthesia, an abdominal incision was made along the ventral midline to expose the uterus and ovaries. The uterus was examined for implantation sites and abnormal tissues. Fetuses were removed, and the numbers of implantation sites, resorptions, live fetuses, and dead fetuses were recorded. In addition, fetuses were also examined for external malformations.

Odd-numbered fetuses were fixed in Bouin’s solution for 2 weeks and subsequently sectioned manually for visceral examination under a stereomicroscope equipped with a hole-plate imaging analysis system. Even-numbered fetuses were eviscerated and immersed in anhydrous ethanol for 2 weeks. Subsequently, the specimens were treated with 1% potassium hydroxide solution to remove the skin and remaining viscera, rendering the muscles transparent, then stained with 0.003% Alizarin Red solution, and examined for skeletal abnormalities under a stereomicroscope.

### 2.7. Non-Targeted Metabolomic Analysis

Metabolite Extraction: Serum samples (100 μL) were mixed with prechilled 80% methanol, vortexed, incubated on ice for 5 min, and then centrifuged at 15,000× *g* and 4 °C for 20 min. The resulting supernatant was diluted with liquid chromatography–mass spectrometry-grade water to a final methanol concentration of 53%, transferred to a new Eppendorf tube, and centrifuged again under the same conditions. The final supernatant was subjected to liquid chromatography–tandem mass spectrometry (LC–MS/MS) analysis.

Ultra-High-Performance Liquid Chromatography Coupled with Tandem Mass Spectrometry (UHPLC-MS/MS) Analysis: Metabolomic analysis was performed using a ThermoFisher Vanquish UHPLC system coupled to an Orbitrap Q Exactive^TM^ HF mass spectrometer (Thermo Fisher Scientific, Bremen, Germany). Chromatographic separation was achieved on a Hypersil Gold column (Thermo Fisher Scientific, Waltham, MA, USA) using a 12 min gradient elution program. For positive ionization mode, the mobile phases consisted of 0.1% formic acid in water (A) and methanol (B). For negative ionization mode, the mobile phases comprised 5 mM ammonium acetate (pH 9.0) (A) and methanol (B). The gradient program was set as follows: 2% B, 1.5 min; 2–85% B, 3 min; 85–100% B, 10 min; 100–2% B, 10.1 min; and 2% B, 12 min. The gradient and mass spectrometer settings were adjusted accordingly for optimal detection. The Q Exactive^TM^ HF mass spectrometer (Thermo Fisher Scientific, Bremen, Germany) was operated in positive/negative ionization mode with a spray voltage of 3.5 kV, capillary temperature of 320 °C, sheath gas flow rate of 35 psi, auxiliary gas flow rate of 10 L/min, S-lens RF level of 60, and auxiliary gas heater temperature of 350 °C.

Data Processing and Statistical Analysis: Raw UHPLC-MS/MS data were processed using Compound Discoverer 3.3 (Thermo Fisher Scientific, Waltham, MA, USA) for peak alignment, peak picking, and quantification. Metabolite identification and quantification were performed by aligning spectral features to databases such as mzCloud, mzVault, and MassList. According to the Metabolomics Standards Initiative framework, metabolites were categorized into five confidence levels: Level 1, confirmed structure by authentic standards with matching retention time and MS/MS spectra in mzVault; Level 2, putative structure based on high-resolution MS/MS spectra from mzCloud or mzVault; Level 3, tentative candidates with multiple possibilities; Level 4, molecular formula assigned by MassList; and Level 5, exact mass features only. Statistical analysis was performed in R v3.4.3 (R Foundation for Statistical Computing, Vienna, Austria), Python v2.7.6 (Python Software Foundation, Beaverton, OR, USA), and CentOS.

Normalization and filtering were performed based on specific criteria to ensure the reliability of the results. Compounds with CVs of relative peak areas exceeding 30% in QC samples were excluded to finalize the identification and relative quantification of the metabolites.

Metabolite Annotation: Metabolites were annotated using the KEGG database (https://www.genome.jp/kegg/pathway.html, accessed on 20 August 2022), HMDB database (https://hmdb.ca/metabolites, accessed on 20 August 2022), and LIPIDMaps database (http://www.lipidmaps.org/, accessed on 20 August 2022). PCA and partial least squares discriminant analysis (PLS-DA) were performed using metaX software (v1.6.0, http://metax.genomics.cn). Univariate analysis, specifically *t*-tests, was employed to determine the statistical significance (P-value) of metabolite variations. Metabolites with variable importance in projection (VIP) > 1, *p* < 0.01, and FC > 2 or < 0.5 were classified as differential metabolites. Volcano plots and hierarchical clustering heat maps were generated to visualize the log_2_(FC) and correlation between differential metabolites. Functional annotation and pathway enrichment analyses were conducted using the KEGG database. Pathways satisfying the enrichment criterion of x/n > y/N and *p* < 0.01 were considered significantly enriched.

### 2.8. Statistical Analysis

Data were assessed for normality and variance homogeneity prior to statistical analysis. Normally distributed continuous variables were expressed as mean ± SD, whereas those with a skewed distribution were presented as the median and interquartile range (IQR). Categorical variables were expressed as frequencies and percentages. For comparing continuous variables across groups, one-way ANOVA was utilized for normally distributed variables, including serum SOD, MDA, GSH, FSH, LH, and E_2_ levels. The Kruskal–Wallis test was applied to skewed data (non-targeted metabolomics analysis), and the Pearson chi-square test was employed to analyze categorical variables (incidence of visceral malformations and delayed skeletal development). All statistical tests were two-sided, and *p* < 0.05 was considered statistically significant. Data analysis and visualization were conducted using SPSS v26.0 (IBM Corp., Armonk, NY, USA), R v3.4.3 (R Foundation for Statistical Computing), Python v2.7.6 (Python Software Foundation), and Prism v8 (GraphPad Software, San Diego, CA, USA).

## 3. Results

### 3.1. Effects of Repeated Diquat Exposure During Pregnancy on Serum Sex Hormone Levels in Female Rats

**Figure 3:**
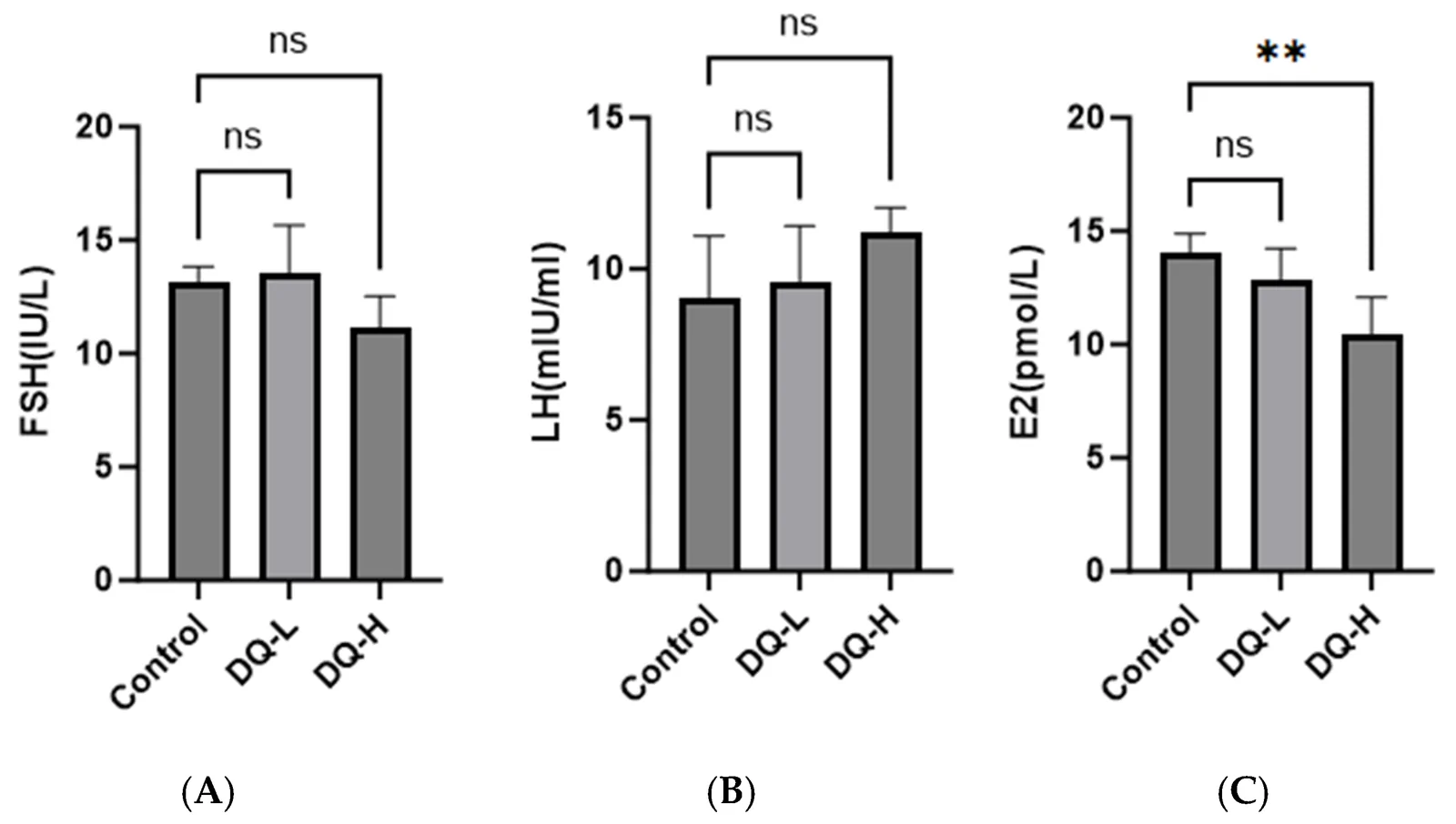
Changes in serum sex hormones following repeated diquat (DQ) exposure during pregnancy. DQ-L and DQ-H represent the low-dose and high-dose DQ exposure groups, respectively. (**A**) Serum follicle-stimulating hormone (FSH) levels following repeated DQ exposure during pregnancy. (**B**) Serum luteinizing hormone (LH) levels following repeated DQ exposure during pregnancy. (**C**) Serum estradiol (E_2_) levels following repeated DQ exposure during pregnancy. Data are expressed as mean ± standard deviation (SD) and were analyzed using one-way analysis of variance. ns indicates no statistically significant difference, ** *p* < 0.01 compared with the control group.

To investigate the effects of repeated DQ exposure during pregnancy on serum sex hormone levels, the serum concentrations of follicle-stimulating hormone (FSH), luteinizing hormone (LH), and E_2_ in pregnant rats were measured using ELISA. One-way analysis of variance (ANOVA) revealed no significant differences in serum FSH and LH levels among the experimental groups (Figure 3; *p* > 0.05). In contrast, serum E_2_ levels were significantly reduced in the high-dose group when compared with the control group (*p* = 0.0014; Table S1). No statistically significant difference in E_2_ levels was noted between the low-dose and high-dose groups (*p* > 0.05).

### 3.2. Effects of Diquat Exposure on Serum Malondialdehyde, Superoxide Dismutase, and Glutathione Levels

**Figure 4:**
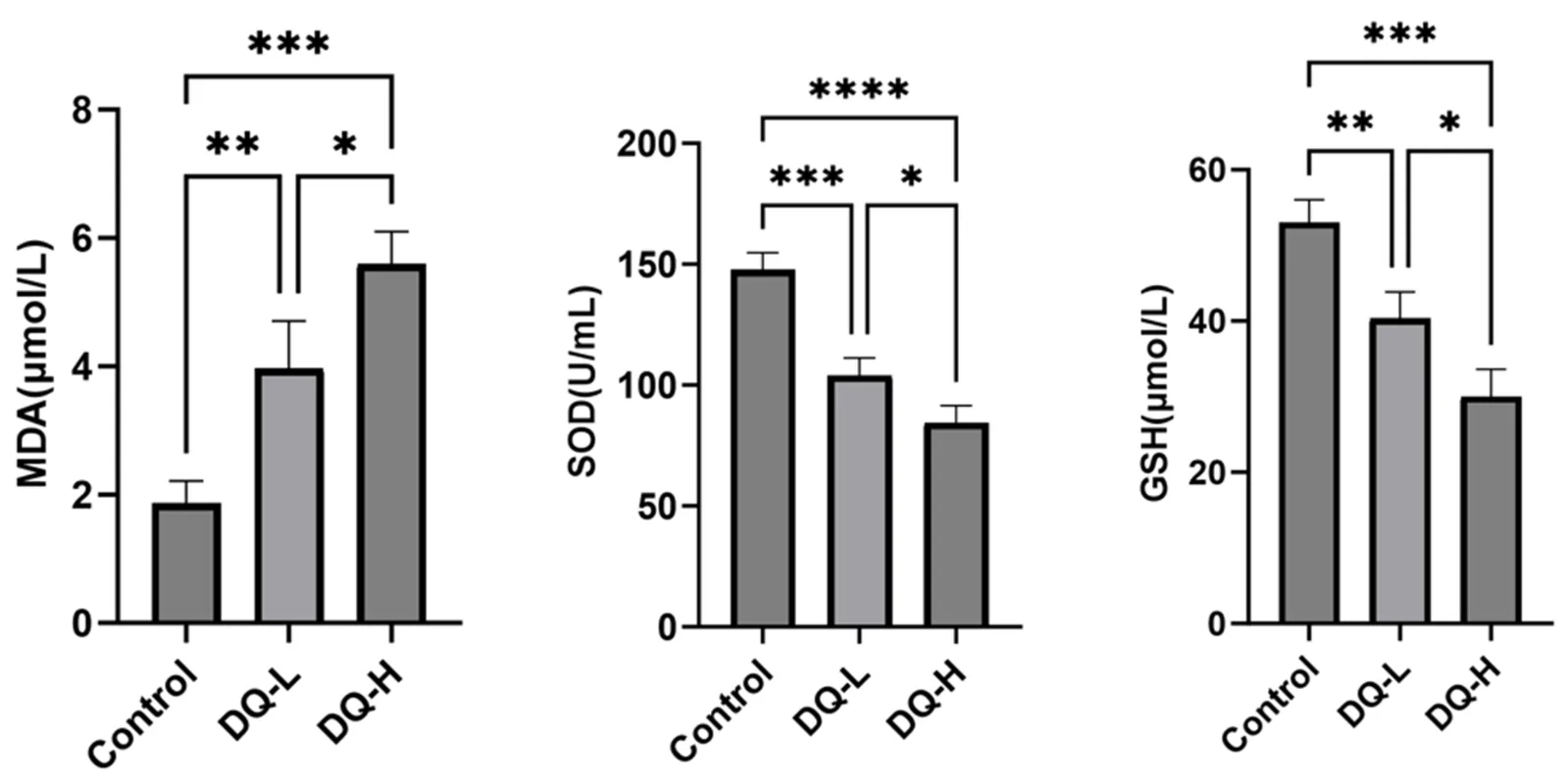
Serum levels of malondialdehyde, superoxide dismutase, and glutathione in the control and diquat-exposed groups. Data are presented as mean ± standard deviation (SD). * *p* < 0.05, ** *p* < 0.01, *** *p* < 0.001, and **** *p* < 0.0001 compared with the control group.

As shown in Figure 4, both the DQ exposure groups exhibited significantly higher serum malondialdehyde (MDA) levels and significantly reduced serum superoxide dismutase (SOD) and glutathione (GSH) levels when compared with the control group (Table S1).

### 3.3. Effects of Diquat Exposure on Reproductive Performance and Fetal Morphology

#### 3.3.1. Reproductive Performance of Pregnant Rats

There were no statistically significant differences among the groups in the rates of resorbed fetuses, live fetuses, and dead fetuses (*p* > 0.05; Table 1).

#### 3.3.2. Repeated Diquat Exposure During Pregnancy Delays Fetal Skeletal Development in Rats

**Figure 5:**
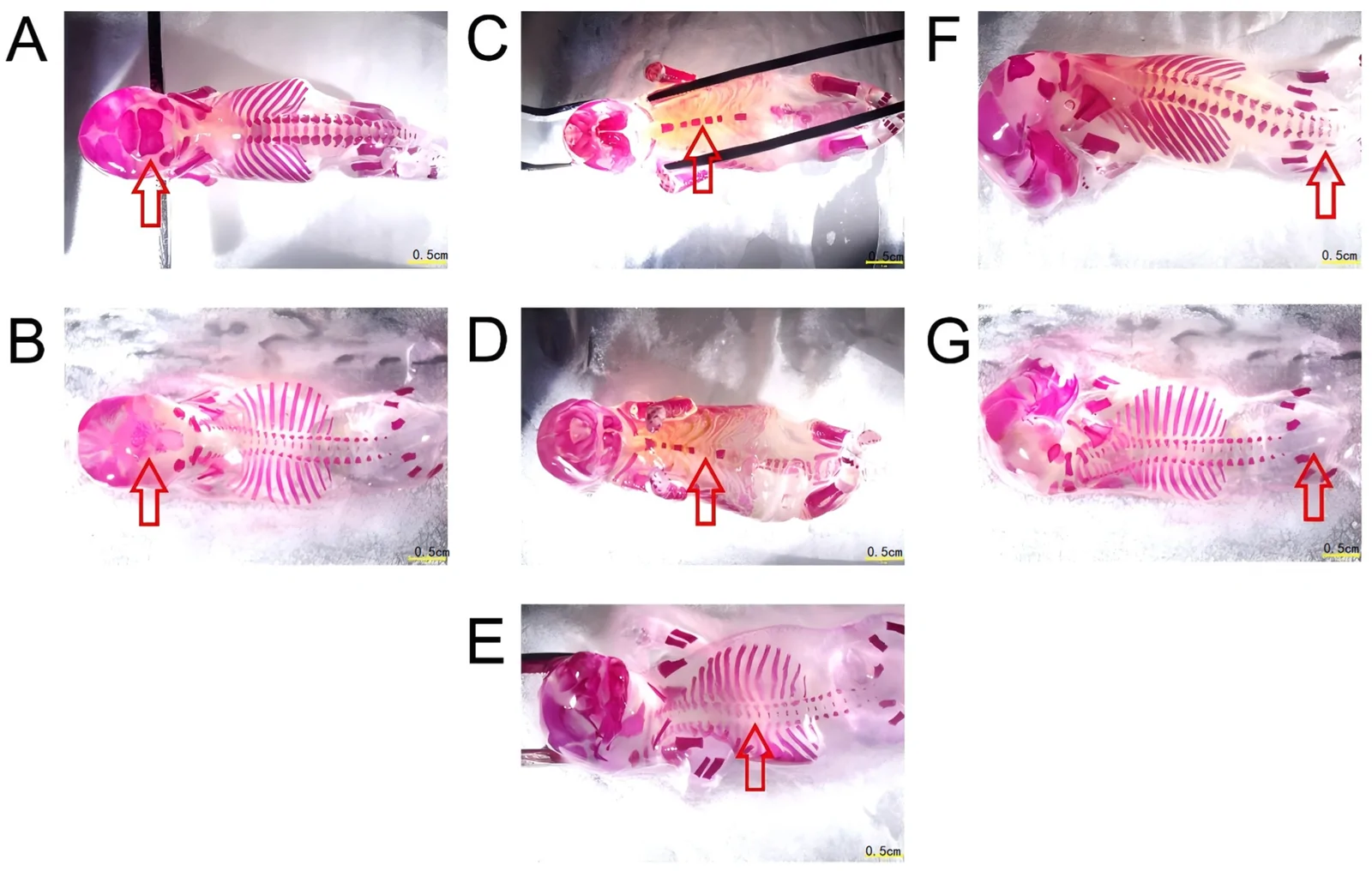
Representative skeletal abnormalities in rat fetuses following repeated diquat exposure during pregnancy. (**A**) Complete ossification of the occipital bone. (**B**) Incomplete ossification of the occipital bone. (**C**) Complete ossification of the sternum. (**D**) Incomplete or absence of ossification of the 5th sternum. (**E**) Absence of sternal ossification. (**F**) Complete ossification of the pelvis. (**G**) Absence of pelvic ossification.

Following Alizarin Red staining of fetal skeletons, significant delays in skeletal development were observed in both the low-dose and high-dose DQ exposure groups. The skeletal anomalies included incomplete ossification of the occipital bone (Figure 5B) as well as absence of ossification of the 5th sternum (Figure 5D), entire sternum (Figure 5E), and caudal vertebrae and pelvic bones (Figure 5G). Despite these skeletal alterations, dissection of the fetuses revealed no apparent external malformations across the groups. Furthermore, microscopic examination of the head sections, along with evaluation of the size, morphology, and positioning of internal organs, revealed no significant deviations when compared with the control group (Table 2).

### 3.4. Alterations in Maternal Serum Metabolites Following Repeated Diquat Exposure During Pregnancy

As shown in Figure S1, Pearson correlation coefficients among quality control (QC) samples exceeded 0.99, indicating high reliability of the obtained metabolomics data. A total of 504 metabolites were confidently identified in negative ionization mode and 714 metabolites were detected in positive ionization mode. Principal component analysis (PCA) did not clearly distinguish the metabolite profiles among the experimental groups (Figure S2). However, PLS-DA scatter plots demonstrated clear separation of metabolite profiles between the groups (Figure 5A). Model validation using 200 permutation tests (Figure S3) revealed satisfactory model robustness and predictive performance. High R^2^ (explained variance) and Q^2^ (predictive ability) values were obtained for each group, as evidenced by Q^2^ intercepts below zero on the Y-axis (Low-dose vs. control: R^2^ = 0.99, Q^2^ = 0.61; High-dose vs. control: R^2^ = 0.99, Q^2^ = 0.58; High-dose vs. low-dose: R^2^ = 0.99, Q^2^ = 0.70).

**Figure 6:**
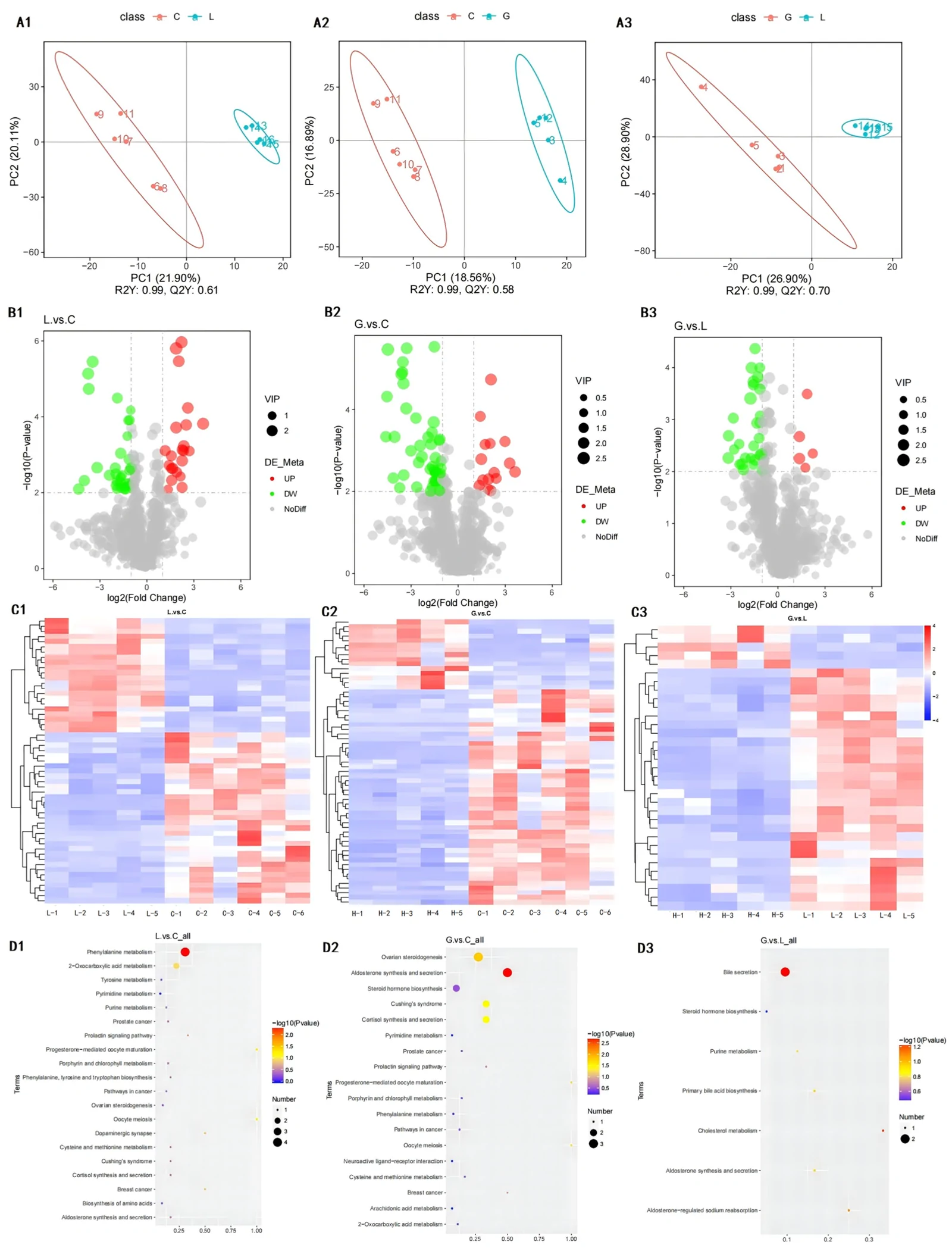
Changes in maternal serum metabolomic profiles following repeated diquat exposure during pregnancy. (**A**) Partial least squares discriminant analysis score plots: (**A1**), low-dose group vs. control group; (**A2**), high-dose group vs. control group; (**A3**), high-dose group vs. low-dose group. (**B**) Volcano plots of differential metabolites: (**B1**), low-dose group vs. control group; (**B2**), high-dose group vs. control group; (**B3**), high-dose group vs. low-dose group. (**C**) Heat maps of differential metabolites: (**C1**), low-dose group vs. control group; (**C2**), high-dose group vs. control group; (**C3**), high-dose group vs. low-dose group. (**D**) KEGG pathway enrichment bubble plots: (**D1**), low-dose group vs. control group; (**D2**), high-dose group vs. control group; (**D3**), high-dose group vs. low-dose group.

Differential metabolite analysis using the criteria VIP > 1.0, fold change (FC) > 2 or FC < 0.5, and *p* < 0.01 identified significant changes among the groups. When compared with the control group, 33 differential metabolites were identified in the low-dose group, including 13 upregulated and 20 downregulated metabolites (Table S2; Figure 6(B1)). In the high-dose group, 43 metabolites were differentially expressed, including seven upregulated and 36 downregulated metabolites, when compared with the control group (Table S3; Figure 6(B2)). Comparison of the high-dose and low-dose groups revealed 22 differential metabolites, including two upregulated and 20 downregulated metabolites (Table S4; Figure 6(B3)). Hierarchical clustering analysis further underscored significant metabolic patterns among the groups (Figure 6C).

Kyoto Encyclopedia of Genes and Genomes (KEGG) pathway enrichment analysis identified key metabolic pathways affected by DQ exposure. In the low-dose group, phenylalanine metabolism was significantly enriched (Figure 6(D1)). In the high-dose group, aldosterone synthesis and secretion and ovarian steroidogenesis were the most prominently affected pathways (Figure 6(D2)). Comparison of the low-dose and high-dose groups revealed significant enrichment of pathways related to bile secretion, aldosterone synthesis and secretion, and cholesterol metabolism (Figure 6(D3)). The differential metabolites closely associated with the toxicological mechanism of DQ are presented in Table 3 and Table 4. In particular, several metabolites were commonly altered in both the exposure groups, when compared with the control group, mainly involving ovarian steroidogenesis, bile secretion, and phenylalanine metabolism (Table 5). Collectively, these findings demonstrate that repeated DQ exposure during pregnancy induced significant changes in maternal serum metabolites and associated metabolic pathways.

## 4. Discussion

In preliminary investigations, DQ exposure was initiated immediately after female rats were co-housed with male rats. This periconceptional exposure regimen resulted in complete pregnancy failure in all treated female rats. To ensure viable pregnancies for developmental toxicity assessment, DQ administration was postponed until gestational day 9 (GD9). GD9 was selected because implantation is complete and embryonic organogenesis has commenced by this stage, allowing evaluation of post-implantation developmental toxicity. A repeated-dose exposure protocol was employed to better mimic sustained physiologically relevant toxicological exposure.

In the present study, maternal serum MDA levels were significantly increased, whereas GSH and SOD activities were significantly decreased following repeated DQ exposure. These findings indicate that DQ induces systemic oxidative stress in pregnant rats. As a major endogenous antioxidant, GSH plays a crucial role in maintaining cellular redox homeostasis [27]. Exposure to DQ is known to generate excessive reactive oxygen species, resulting in oxidative stress, significant depletion of endogenous antioxidant defenses such as SOD and GSH, and considerable accumulation of lipid peroxidation products such as MDA [28,29,30]. The observed elevation of MDA and depletion of SOD and GSH are consistent with the known mechanism of diquat toxicity, which generates superoxide anions through redox cycling and disrupts glutathione metabolism [5] https://ngdc.cncb.ac.cn/openlb/publication/OLB-PM-26785375, accessed on 22 July 2026. Importantly, oxidative stress is not merely a correlative biomarker but a mechanistic driver of impaired osteogenesis: it interferes with osteoblast differentiation, enhances adipocyte formation from mesenchymal stem cells, and stimulates osteoclast activity, leading to bone resorption and reduced mineralization [31] https://www.sciencedirect.com/science/article/pii/S2667009725000508, accessed on 22 July 2026. This mechanistic framework, supported by recent evidence on pollutant-induced oxidative stress and skeletal outcomes [32,33], strongly suggests that the redox imbalance we detected underlies the fetal developmental toxicity observed in this study.

Although inflammatory cytokines were not directly measured in this study, the observed oxidative stress strongly suggests a potential inflammatory component. Diquat is known to activate the TLR4/MyD88/NF-κB and MAPK signaling pathways via ROS generation, leading to elevated TNF-α, IL-6, and IL-1β [34,35]. Given the crosstalk between oxidative stress and inflammation in developmental toxicity, it is plausible that similar inflammatory responses contribute to the skeletal ossification deficits observed in our study. This hypothesis warrants further investigation.

Maternal serum E_2_ levels on GD19 were significantly decreased following repeated DQ exposure, whereas LH and FSH remained unchanged. This uncoupled hormonal profile points to peripheral (placental/ovarian) endocrine dysfunction rather than primary ovarian insufficiency, likely via direct inhibition of CYP19A1, while the lack of compensatory LH/FSH elevation may reflect pituitary toxicity or placental independence from gonadotropin regulation [36,37]. The ovary is a plausible additional target: DQ-induced oxidative stress can impair granulosa cell function and reduce aromatase expression [5], consistent with our metabolomic finding of “ovarian steroidogenesis” perturbation (progesterone accumulation with reduced E_2_). Given that maternal E_2_ is essential for fetal endochondral ossification—regulating chondrocyte proliferation, osteoblast differentiation, and mineralization [22]—this steroidogenic disruption likely underlies the 100% incidence of ossification deficits. However, this mechanism remains inferential without direct enzymatic or receptor-level validation.

Notably, DQ exposure sharply increased the incidence of incomplete ossification sites from 16.7% (only sternal variations) to 100%, indicating marked impairment of fetal skeletal development. The fetal toxicity observed in this study is mechanistically supported by evidence that diquat crosses the placenta. Bus et al. (1975) demonstrated that after maternal administration of ^14^C-diquat to pregnant rats, radioactivity was consistently detected in whole fetuses, with fetal concentrations exceeding those of paraquat, correlating with greater fetal toxicity [38]. Fetal exposure likely occurs through direct placental transfer and potentially via accumulation in the amniotic fluid, as documented for other bipyridyl compounds [38,39]. Thus, the severe skeletal ossification delays observed in our study are consistent with direct fetal exposure to diquat during the critical period of endochondral ossification. Moreover, the observed delays in skeletal ossification may be associated with the reduction in maternal E_2_ levels, given the established role of estrogen signaling in fetal bone development [22]. A critical unresolved question is whether the observed ossification deficits represent a transient developmental delay or a permanent skeletal malformation. The current study, with termination at GD20, captures a single developmental time point and cannot distinguish between these possibilities.

Furthermore, although no significant external or visceral malformations were found in the fetuses, both the DQ exposure groups exhibited pronounced delays in skeletal development, characterized by incomplete ossification of the occipital bone, sternum, pelvic bone, and caudal vertebrae, with relatively high incidence rates. Previous studies have reported that paraquat exposure reduces fetal weight and delays ossification. Bus et al. [38] indicated that DQ increased the incidence of sternum defects, while Selypes et al. [7] observed skeletal abnormalities in DQ-exposed mouse fetuses, including larger zygomatic bones, wider brain structures, flat and dumbbell-shaped vertebral bodies, missing or incomplete ossification points in the sternum, missing ossification nuclei in the metatarsal and phalangeal bones, delayed skeletal development, and osteoporosis. Similarly, Sewalk et al. [10] reported brain, eye, oral, limb, and pelvic malformations, skeletal scoliosis, and incomplete ossification in DQ-exposed fetuses. Collectively, these findings demonstrate the developmental toxicity of DQ and indicate that the fetal skeleton is a sensitive target of gestational DQ exposure.

Metabolomic analysis further identified alterations in pathways related to ovarian steroidogenesis, phenylalanine metabolism, and cholesterol metabolism, suggesting that disruption of these pathways may contribute to the developmental toxicity of DQ. It is important to acknowledge that the metabolomics findings presented here are derived from an untargeted screening approach, which is inherently discovery-oriented rather than confirmatory. While pathway enrichment analysis revealed several perturbed metabolic routes, it does not by itself establish causality. However, the biological coherence of these pathways with our observed phenotypic effects—namely, the suppression of steroidogenesis, elevation of oxidative stress, and fetal skeletal anomalies—lends strong internal validity to the results. Notably, previous studies on pollutant-induced metabolomic alterations have similarly identified overlapping pathways, supporting the sensitivity and relevance of metabolomics for toxicity screening [40]. Nevertheless, we acknowledge that targeted metabolomic quantification of key differential metabolites and functional enzymatic assays are required to definitively validate the mechanisms proposed here. Such validation studies are a priority for our ongoing work.

It is essential to contextualize the doses employed in this study: although derived from a fraction of the LD_50_, they exceed typical environmental residue levels. Consequently, direct extrapolation of these findings to environmentally relevant low-dose exposure scenarios warrants caution. This design was intentionally chosen to amplify the toxicological response, enabling a clear and statistically robust elucidation of diquat’s impact on placental steroidogenesis, maternal metabolism, and fetal skeletal development. The high incidence (100%) of developmental effects observed provides a definitive evidence base for the teratogenic potential of diquat. The identified pathways—oxidative stress, disruption of ovarian steroidogenesis, and altered phenylalanine and aldosterone metabolism—are biologically conserved and thus offer a mechanistic framework relevant to human health risk assessment. However, we acknowledge that future studies using environmentally relevant, lower doses over prolonged exposure periods are essential to define the no-observed-adverse-effect level (NOAEL) and to fully characterize the dose–response relationship for chronic exposure scenarios.

Several limitations of this study should be acknowledged. First, regarding the metabolomics analysis, while permutation tests were performed to assess PLS-DA model validity, the modest sample size (*n* = 5–6 per group) renders supervised multivariate models susceptible to overfitting. Therefore, the metabolic pathways identified in this study—including ovarian steroidogenesis, phenylalanine metabolism, and aldosterone synthesis—should be interpreted as exploratory and hypothesis-generating rather than confirmatory. The biological coherence of these pathways with our independent phenotypic endpoints (e.g., reduced E_2_, oxidative stress, skeletal deficits) lends some internal validity to the findings; however, targeted metabolomic quantification in larger, independent cohorts is essential to validate these candidate pathways and establish robust mechanistic links. Second, the study primarily relied on endpoint measurements of serum metabolites and hormone levels. Given that serum metabolomics reflects systemic rather than tissue-specific alterations, the identified pathways may not precisely mirror events occurring in target organs such as the ovary or placenta. Mechanistic validation in these tissues—including steroidogenic enzyme activity (e.g., CYP19A1), estrogen receptor expression, and tissue-specific oxidative stress markers—was not performed. Third, histological examination of maternal ovaries, placenta, and fetal tissues was not conducted, as all fetal samples were used for skeletal evaluations. Fourth, serum E_2_ levels were measured solely by ELISA, without LC–MS/MS confirmation. Finally, as the current study terminated at GD20, we cannot distinguish between transient developmental delay and permanent skeletal malformation; postnatal follow-up studies are required to determine the permanency of these effects. Consequently, the proposed mechanism remains correlative, and causal relationships require confirmation through subsequent targeted mechanistic studies.

## 5. Conclusions

This study provides preliminary evidence that gestational DQ exposure is associated with oxidative stress, endocrine perturbation, and fetal skeletal ossification deficits in rats. The metabolomic data offer valuable hypothesis-generating insights into potential metabolic pathways that may contribute to these effects, but causal relationships remain to be established. These findings should be interpreted as a foundation for future mechanistic investigations rather than as definitive evidence of a complete toxicological pathway.

## Figures and Tables

**Table 1:** Effects of repeated diquat exposure during pregnancy on reproductive performance and fetal development in rats.

Groups	Implantation Number/Litter	Number of Resorbed Fetuses (%)	Number of Live Fetuses (%)	Number of Dead Fetuses (%)
Control	58/6	0 (0)	58 (100)	0 (0)
DQ-L	59/5	2 (3.4)	57 (96.6)	0 (0)
DQ-H	72/5	0 (0)	72 (100)	0 (0)

**Table 2:** Skeletal anomalies in rat fetuses following repeated diquat exposure during pregnancy.

Examination Indicators	Control	Low-Dose Diquat (DQ-L)	High-Dose Diquat (DQ-H)
Number of litters examined	6	5	5
Incomplete ossification of occipital bone			
Number of affected litters	0	5	5
Litter incidence (%)	0	100	100
Incomplete ossification/absence of sternum			
Number of affected litters	1	5	5
Litter incidence (%)	16.7	100	100
Total number of litters with incomplete ossification	1	5	5
Total litter incidence of incomplete ossification (%)	16.7	100	100

**Table 3:** Significantly altered metabolites identified between the low-dose diquat exposure group and control group (L vs. C).

No.	Metabolite	The Level of Identification	FC	*p*-Value	ROC	VIP	Trends	Metabolic Pathway
1	Progesterone	2	4.63	1.09 × 10^−6^	1	2.59	up	Steroid hormone biosynthesis; Ovarian steroidogenesis; Aldosterone synthesis and secretion
2	Hippuric acid	2	0.07	7.30 × 10^−6^	1	2.45	down	Phenylalanine metabolism
3	Phenylacetaldehyde	2	4.76	1.44 × 10^−3^	1	2.36	up	Phenylalanine metabolism
4	Stercobilin	2	0.19	3.47 × 10^−3^	0.93	2.04	down	Porphyrin and chlorophyll metabolism
5	2-Keto-4-methylthiobutyric acid	2	0.06	4.81 × 10^−3^	0.97	2.07	down	Cysteine and methionine metabolism
6	Lithocholic acid	2	0.31	7.35 × 10^−3^	0.97	1.20	down	Bile secretion

**Table 4:** Significantly altered metabolites identified between the high-dose diquat exposure group and control group (H vs. C).

No.	Metabolite	The Level of Identification	FC	*p*-Value	ROC	VIP	Trends	Metabolic Pathway
1	Lithocholic acid	2	0.04	3.46 × 10^−6^	1	2.57	down	Bile secretion
2	Hippuric acid	2	0.09	7.00 × 10^−6^	1	2.15	down	Phenylalanine metabolism
3	Glycoursodeoxycholic acid	2	0.04	4.64 × 10^−4^	1	2.02	down	Primary bile acid biosynthesis
4	(±)15-HETE	2	0.44	5.38 × 10^−4^	1	2.16	down	Arachidonic acid metabolism
5	Progesterone	2	3.30	7.16 × 10^−4^	1	1.80	up	Steroid hormone biosynthesis; ovarian steroidogenesis; aldosterone synthesis and secretion
6	Stercobilin	2	0.12	1.02 × 10^−3^	1	2.03	down	Porphyrin and chlorophyll metabolism
7	Propionylcarnitine	2	2.78	1.63 × 10^−3^	1	2.22	up	Lipid metabolism; branched-chain amino acid degradation
8	(9cis)-Retinal	2	0.42	4.44 × 10^−3^	1	2.13	down	retinol metabolism
9	2-Keto-4-methylthiobutyric acid	2	0.06	4.71 × 10^−3^	1	1.96	down	Cysteine and methionine metabolism; 2-Oxocarboxylic acid metabolism
10	Uridine	2	0.50	8.88 × 10^−3^	0.93	1.91	down	Pyrimidine metabolism
11	Pregnenolone	2	0.30	9.75 × 10^−3^	1	2.08	down	Steroid hormone biosynthesis; ovarian steroidogenesis; aldosterone synthesis and secretion

**Table 5:** Common differential metabolites in the low-dose and high-dose diquat exposure groups compared with the control group (L vs. C and H vs. C).

No.	Metabolite	L vs. C					Metabolic Pathway	G vs. C				
		FC	*p*-Value	ROC	VIP	Trends		Trends	ROC	VIP	*p*-Value	FC
1	Progesterone	4.63	1.09 × 10^−6^	1	2.55	↑	Aldosterone synthesis and secretion; Ovarian steroidogenesis; Cortisol synthesis and secretion	↑	1	1.77	7.16 × 10^−4^	3.3
2	Lithocholic acid	0.31	7.35 × 10^−3^	0.97	1.20	↓	Bile secretion	↓	1	2.53	3.46 × 10^−6^	0.04
3	Octopamine	0.08	1.84 × 10^−5^	1	2.39	↓	Neuroactive ligand-receptor interaction	↓	1	2.13	1.24 × 10^−5^	0.09
4	Hippuric acid	0.07	7.30 × 10^−6^	1	2.42	↓	Phenylalanine metabolism	↓	1	2.12	7.00 × 10^−6^	0.09

## Data Availability

The raw data supporting the conclusions of this article will be made available by the authors on request.

## Supplementary Materials

The following supporting information can be downloaded at: https://www.mdpi.com/article/10.3390/toxics14080656/s1. Figure S1: Pearson correlation between QC samples. Figure S2: PCA distribution of altered serum metabolic profiles in pregnant rats induced by repeated exposure to diquat during pregnancy. (a) PCA distribution map of the control group vs. the low-dose group. (b) PCA-3D distribution map of the control group vs. the low-dose group. (c) PCA distribution map of the control group vs. the high-dose group. (d) PCA-3D distribution map of the control group vs. the high-dose group. (e) PCA distribution map of the low-dose group vs. the high-dose group. (f) PCA-3D distribution map of the low-dose group vs. the high-dose group. Figure S3: Validation plot of PLS-DA model sequencing for changes in serum metabolic profiles in pregnant rats induced by repeated exposure to diquat during pregnancy. (a) Low-dose group vs. control group. (b) High-dose group vs. control group. (c) High-dose group vs. low-dose group. Table S1: The contents of different indices (FSH, LH, E2, MDA, SOD, GSH). Table S2: Significant differential metabolites between low-dose and control groups. Table S3: Significant differential metabolites between high-dose and control groups. Table S4: Significant differential metabolites between high-dose and low-dose groups.

## Abbreviations

E_2_: estradiol; LH: luteinizing hormone; FSH: follicle-stimulating hormone; ROS: reactive oxygen species; LD_50_: lethal dose 50; ELISA: enzyme-linked immunosorbent assay; OD: optical density; KOH: potassium hydroxide; PCA: Principal components analysis; PLS-DA: Partial least squares discriminant analysis; FC: fold change; SD: standard deviation; IQR: interquartile range; ANOVA: one-way analysis of variance; HETE: hydroxyeicosatetraenoic acid.
