# Supplementary material for: Serum Metabolomic Alterations and Developmental Toxicity Induced by Diquat Exposure in Pregnant Rats and Their Fetuses: A Preliminary Study
Source: Toxics. 2026 Jul 25;14(8):656. doi: 10.3390/toxics14080656 (PMC13517744; doi:10.3390/toxics14080656)
Supplement: Supplementary file 1 [file toxics-14-00656-s001.zip › toxics-4414690-supplementary.pdf]

Table S1 The contents of different indices (FSH, LH, E2, MDA, SOD, GSH).

Index	Control（average±SD）	L（average±SD）	H（average±SD）
FSH	13.2±0.68	13.5±2.15	11.1±1.43
LH	9.0±2.10	9.6±1.82	11.2±0.84
E2	14±0.89	12.8±1.44	10.4±1.67
MDA	1.87±0.35	3.97±0.74	5.6±0.5
SOD	147.7±7.02	104.0±7.21	84.3±7.23
GSH	53.0±3.0	40.3±3.51	30.0±3.6

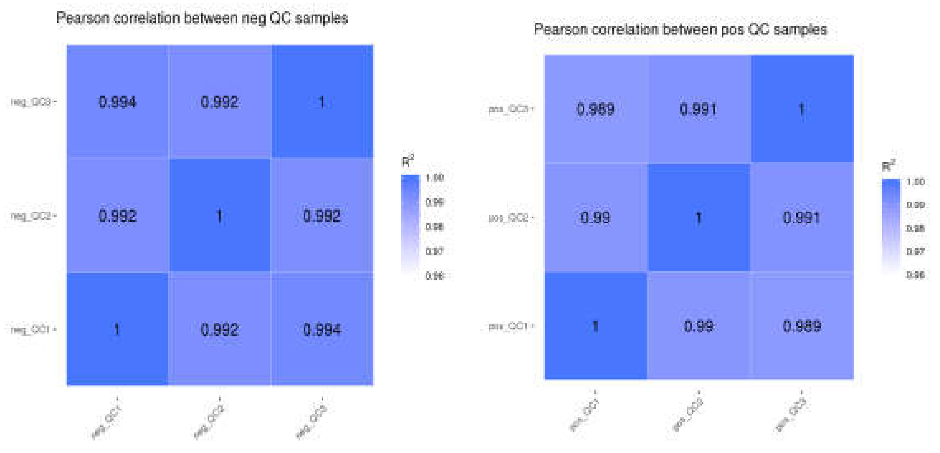

Figure S1: Pearson correlation between QC samples

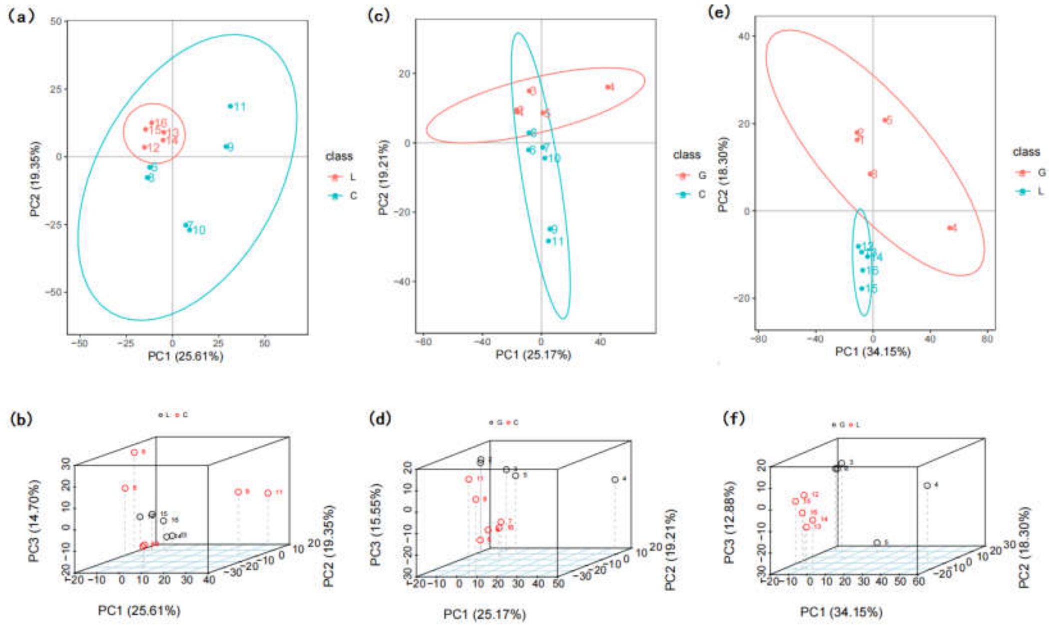

Figure S2. PCA distribution of altered serum metabolic profiles in pregnant rats induced by repeated exposure to diquat during pregnancy. (a) PCA distribution map of the control group vs. the low-dose group. (b) PCA-3D distribution map of the control group vs. the low-dose group. (c) PCA distribution map of the control group vs. the high-dose group. (d) PCA-3D distribution map of the control group vs. the high-dose group. (e) PCA distribution map of the low-dose group vs. the high-dose group. (f) PCA-3D distribution map of the low-dose group vs. the high-dose group.

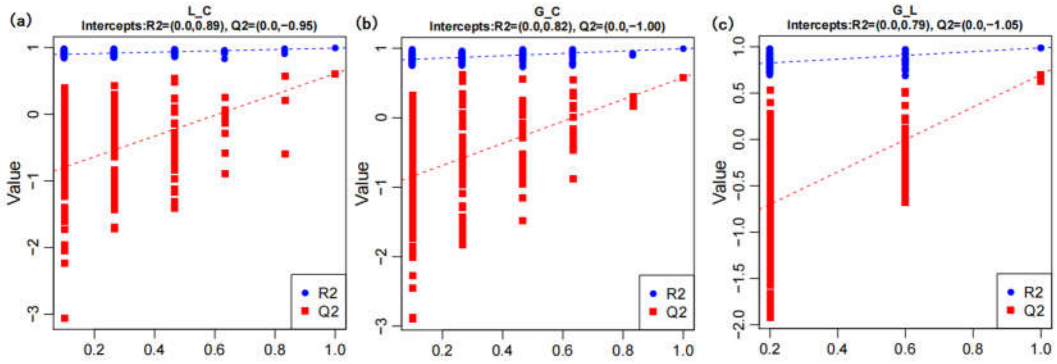

Figure S3. Validation plot of PLS-DA model sequencing for changes in serum metabolic profiles in pregnant rats induced by repeated exposure to diquat during pregnancy. (a) Low-dose group vs. control group. (b) High-dose group vs. control group. (c) High-dose group vs. low-dose group.

Tabel S2

Significant differential metabolites between low-dose and control groups

Name	The level of identification	mzCloud_Results	mzVault_Results	MassList_Results	FC	log2FC	Pvalue	ROC	VIP	Up.Down
Progesterone	2	Full match	Full match	Full match	4.63	2.21	1.09E-06	1	2.59	up
Deoxyinosine	4	No results	No results	Full match	3.65	1.87	1.58E-06	1	2.78	up
Hippuric acid	2	Full match	Full match	Full match	0.07	-3.75	7.30E-06	1	2.45	down
Octopamine	4	No results	No results	Full match	0.08	-3.72	1.84E-05	1	2.43	down
L-Threonic acid	2	Full match	Full match	No results	0.49	-1.03	6.70E-05	1	1.26	down
Phenylglyoxylic acid	4	No results	No results	Full match	5.70	2.51	1.64E-04	1	2.26	up
Phenylpyruvic acid	4	No results	No results	Full match	4.80	2.26	7.46E-04	1	2.15	up
Taurochenodeoxycholic Acid (sodium salt)	5	Invalid mass	No results	No results	5.08	2.34	7.92E-04	1	1.95	up
Taurolithocholic acid	4	No results	No results	Full match	6.57	2.72	8.01E-04	0.97	1.75	up
N-Acetyl-L-tyrosine	2	Full match	Full match	Full match	0.31	-1.67	9.17E-04	1	2.09	down
3-Indoxyl sulphate	2	Full match	Full match	Full match	2.95	1.56	1.07E-03	1	1.96	up
2,3,4-Trihydroxybenzoic acid	4	No results	No results	Full match	0.46	-1.13	1.32E-03	1	2.00	down
Phenylacetaldehyde	2	Full match	No results	No results	4.76	2.25	1.44E-03	1	2.36	up
Indoxylsulfuric acid	4	No results	No results	Full match	2.67	1.41	1.90E-03	1	1.88	up
2-Deoxyuridine	4	No results	No results	Full match	0.50	-1.01	2.24E-03	0.97	2.07	down
Homoarginine	2	No results	Full match	Full match	3.13	1.65	2.28E-03	1	2.10	up
PG 18:0_18:2	2	No results	Full match	No results	2.97	1.57	2.31E-03	1	1.87	up

Dihydrothymine	2	Full match	No results	No results	0.47	-1.10	2.56E-03	0.97	1.76	down
3-Coumaric acid	2	Full match	Full match	Full match	3.75	1.91	2.88E-03	0.97	1.86	up
N-Oleoyl Glycine	4	No results	No results	Full match	0.26	-1.97	3.22E-03	0.93	2.01	down
trans-2-Butene-1,4-dicarboxylic Acid	4	No results	No results	Full match	0.33	-1.58	3.44E-03	0.97	1.39	down
Stercobilin	2	Full match	No results	No results	0.19	-2.40	3.47E-03	0.93	2.04	down
Lysops 22:5	4	No results	No results	Full match	0.32	-1.66	3.90E-03	0.97	1.55	down
2-Keto-4-methylthiobutyric acid	2	Full match	No results	No results	0.06	-3.99	4.81E-03	0.97	2.07	down
L-Methionine Methyl Ester	4	No results	No results	Full match	0.45	-1.15	5.57E-03	0.93	1.15	down
Homovanillic acid	4	No results	No results	Full match	0.25	-1.98	6.41E-03	0.93	1.80	down
Proline-hydroxyproline	2	No results	Full match	No results	0.49	-1.03	7.21E-03	0.87	1.63	down
12-Hydroxydodecanoic acid	2	Full match	No results	No results	0.37	-1.43	7.31E-03	1	1.32	down
Lithocholic acid	2	Full match	No results	Full match	0.31	-1.69	7.35E-03	0.97	1.20	down
Flavin mononucleotide (FMN)	2	Full match	No results	Full match	0.26	-1.92	8.02E-03	0.97	1.51	down
N-Acetyl-D-galactosamine 4-sulfate	2	Full match	No results	No results	0.37	-1.43	8.16E-03	0.97	1.52	down
13,14-Dihydro prostaglandin E1	2	Full match	No results	No results	0.39	-1.35	8.27E-03	1	1.49	down
Taurodeoxycholic acid	5	Invalid mass	No results	No results	2.55	1.35	8.28E-03	1	1.39	up

Tabel S3

Significant differential metabolites between high-dose and control groups

Name	The level of identification	mzCloud_Results	mzVault_Results	MassList_Results	FC	log2FC	Pvalue	ROC	VIP	Up.Down
Lithocholic acid	2	Full match	No results	Full match	0.04	-4.48	3.46E-06	1	2.57	down
Hippuric acid	2	Full match	Full match	Full match	0.09	-3.53	7.00E-06	1	2.15	down
Octopamine	4	No results	No results	Full match	0.09	-3.55	1.24E-05	1	2.16	down
Flavin mononucleotide	2	Full match	No results	Full match	0.09	-3.48	2.33E-05	1	2.36	down
Thymidine 5’-monophosphate	4	Invalid mass	No results	No results	0.04	-4.53	4.87E-05	1	2.06	down
12(S)-HETE	4	No results	No results	Full match	0.34	-1.55	2.05E-04	1	2.33	down
LPE O-20:4	2	No results	Full match	No results	0.32	-1.66	3.64E-04	1	2.09	down
Glycoursodeoxycholic acid	2	Full match	Full match	Full match	0.04	-4.60	4.64E-04	1	2.02	down
N-Acetyl-L-tyrosine	2	Full match	Full match	Full match	0.27	-1.91	4.78E-04	1	2.20	down

(±)15-HETE	3	Full match	No results	Full match	0.44	-1.17	5.38E-04	1	2.16	down
LPS 22:4	2	No results	Full match	No results	0.43	-1.21	5.74E-04	0.97	1.81	down
Progesterone	2	Full match	Full match	Full match	3.30	1.72	7.16E-04	1	1.80	up
Lysops 22:5	4	No results	No results	Full match	0.19	-2.42	7.63E-04	1	2.29	down
Stercobilin	2	Full match	No results	No results	0.12	-3.01	1.02E-03	1	2.03	down
13,14-Dihydro prostaglandin E1	2	Full match	No results	No results	0.20	-2.31	1.43E-03	1	2.28	down
LPS 22:5	2	No results	Full match	Full match	0.37	-1.44	1.51E-03	0.97	2.13	down
Propionylcarnitine	2	Full match	Full match	Full match	2.78	1.48	1.63E-03	1	2.22	up
LPA 22:6	2	No results	Full match	No results	0.47	-1.08	1.81E-03	1	1.85	down
β-Cortolone	5	Invalid mass	No results	No results	0.18	-2.51	1.97E-03	1	2.10	down
LPS 16:0	2	No results	Full match	No results	0.32	-1.62	2.48E-03	0.97	2.11	down
LPS 20:4	2	No results	Full match	No results	0.47	-1.10	2.74E-03	0.97	1.97	down
LPA 18:1	2	No results	Full match	No results	0.29	-1.78	2.86E-03	0.93	2.00	down

LPS 20:5	2	No results	Full match	No results	0.36	-1.49	2.97E-03	1	2.20	down
LPE O-17:1	2	No results	Full match	No results	0.36	-1.47	3.37E-03	0.97	2.03	down
Dihydrothymine	2	Full match	No results	No results	0.38	-1.39	3.49E-03	1	2.07	down
LPI 16:0	2	No results	Full match	No results	0.46	-1.11	3.61E-03	0.97	1.52	down
Lysops 22:6	4	No results	No results	Full match	0.09	-3.47	3.99E-03	1	2.11	down
LPS 18:1	2	No results	Full match	No results	0.44	-1.18	4.09E-03	0.97	1.92	down
(9cis)-Retinal	2	Full match	No results	Full match	0.42	-1.25	4.44E-03	1	2.13	down
LPE O-16:1	2	No results	Full match	No results	0.43	-1.23	4.49E-03	0.93	2.04	down
2-Keto-4-methylthiobutyric acid	2	Full match	No results	No results	0.06	-4.13	4.71E-03	1	1.96	down
Nicotinate ribonucleoside	4	No results	No results	Full match	3.83	1.94	4.99E-03	0.9	2.01	up
LPE O-20:3	2	No results	Full match	No results	0.44	-1.18	5.28E-03	1	1.72	down
α-Hydroxyhippuric acid	2	Full match	No results	No results	2.99	1.58	5.28E-03	1	2.00	up

LPS 22:6	2	No results	Full match	Full match	0.38	-1.40	5.51E-03	1	2.13	down
LPC 21:0-SN1	2	No results	Full match	No results	0.37	-1.44	5.69E-03	0.97	1.96	down
PE 17:1_17:1	2	No results	Full match	No results	2.55	1.35	7.11E-03	0.97	1.88	up
Dodecanedioic acid	2	Full match	No results	No results	0.16	-2.64	7.80E-03	0.97	1.96	down
1a,1b-Dihomo prostaglandin E1	5	Invalid mass	No results	No results	0.08	-3.72	8.13E-03	1	1.92	down
DG 16:0_18:2	5	No results	Invalid mass	No results	3.79	1.92	8.81E-03	0.9	1.73	up
Uridine	2	Full match	Full match	Full match	0.50	-1.00	8.88E-03	0.93	1.91	down
LPC O-17:1	2	No results	Full match	No results	0.44	-1.18	9.59E-03	0.97	1.89	down
Pregnenolone	2	Full match	No results	Full match	0.30	-1.75	9.75E-03	1	2.08	down

Tabel S4. Significant differential metabolites between high-dose and low-dose groups

Name	The level of identification	mzCloud_Results	mzVault_Results	MassList_Results	FC	log2FC	Pvalue	ROC	VIP	Up.Down
LPS 18:0	2	No results	Full match	No results	0.45	-1.15	1.00E-04	1	1.65	down
LPE O-20:4	2	No results	Full match	No results	0.31	-1.68	1.01E-04	1	2.22	down
LPC 19:0-SN1	2	No results	Full match	No results	0.41	-1.28	1.52E-04	1	2.12	down
LPS 22:4	2	No results	Full match	No results	0.34	-1.54	1.87E-04	1	2.38	down
LPA 18:1	2	No results	Full match	No results	0.34	-1.56	2.14E-04	1	1.96	down
LPS 18:1	2	No results	Full match	No results	0.45	-1.15	2.59E-04	1	2.04	down
Taurochenodeoxycholic acid	2	Full match	No results	Full match	0.30	-1.75	3.92E-04	1	2.58	down
Deoxyinosine	4	No results	No results	Full match	0.46	-1.13	8.48E-04	1	1.62	down
1a,1b-Dihomo prostaglandin E1	5	Invalid mass	No results	No results	0.14	-2.84	9.39E-04	1	1.79	down
9-HOTrE	4	No results	No results	Full match	0.45	-1.14	1.14E-03	1	1.71	down
LPE O-20:3	2	No results	Full match	No results	0.40	-1.33	1.17E-03	1	2.09	down
Aldosterone	5	Invalid mass	No results	No results	0.18	-2.47	2.96E-03	0.92	1.54	down
Dehydrocholic acid	5	Invalid mass	No results	No results	0.34	-1.54	3.21E-03	1	1.66	down
Lithocholic acid	2	Full match	No results	Full match	0.15	-2.78	3.76E-03	1	1.51	down

LPE O-15:1	2	No results	Full match	No results	0.49	-1.03	4.55E-03	1	2.11	down
LPE O-17:1	2	No results	Full match	No results	0.45	-1.15	4.82E-03	1	1.74	down
SM 9:1;20/34:3	2	No results	Full match	No results	0.11	-3.16	5.49E-03	1	2.13	down
β-Cortolone	5	Invalid mass	No results	No results	0.30	-1.73	5.50E-03	1	1.60	down
Glu-Gln	2	No results	Full match	No results	2.59	1.37	5.62E-03	1	2.08	up
Taurodeoxycholic acid	5	Invalid mass	No results	No results	0.21	-2.27	7.20E-03	1	2.19	down
Nicotinate ribonucleoside	4	No results	No results	Full match	3.36	1.75	8.44E-03	1	1.35	up
7-Ketolithocholic acid	4	No results	No results	Full match	0.22	-2.19	9.16E-03	1	2.13	down
